# Structural insights into allosteric inhibition of HRI kinase by heme binding via HDX-MS

**DOI:** 10.1042/BCJ20253072

**Published:** 2025-06-17

**Authors:** Shivani Kanta, Vanesa Vinciauskaite, Graham Neill, Miratul M.K. Muqit, Glenn R. Masson

**Affiliations:** 1 Division of Cancer Research, School of Medicine, University of Dundee, Dundee DD1 9SY, U.K; 2 MRC Protein Phosphorylation and Ubiquitylation Unit, School of Life Sciences, University of Dundee, Dundee DD1 5EH, U.K

**Keywords:** hydrogen–deuterium exchange mass spectrometry, iron, protein-serine-threonine kinases, protein-tyrosine kinases, stress kinases, stress response

## Abstract

Heme-regulated inhibitor (HRI) is one of the four mammalian kinases that phosphorylate eIF2α, facilitating a cellular response to stress through the regulation of mRNA translation. Originally identified as a heme sensor in erythroid progenitor cells, HRI has since emerged as a potential therapeutic target in both cancer and neurodegeneration. Here, we characterise two modes of HRI inhibition using structural mass spectrometry, biochemistry, and biophysics. We examined several competitive ATP-mimetic inhibitors – dabrafenib, encorafenib, and GCN2iB – and compared them with the heme-mimetic allosteric inhibitor, hemin. By combining hydrogen–deuterium exchange mass spectrometry with protein models generated by AlphaFold 3, we investigated the structural basis of inhibition by dabrafenib and hemin. Our analysis revealed that hemin inhibition induces large-scale structural rearrangements in HRI, which are not observed with ATP-mimetic inhibitors. Our results suggest that HRI may be inhibited using two distinctly different modalities, which may guide future drug development.

## Introduction

Heme-regulated inhibitor (HRI) was originally identified as a key regulator in erythroid cells, where it activates the integrated stress response (ISR) in response to heme deprivation during terminal erythropoiesis [1,2]. This activation facilitates the balanced translation of alpha-globin and beta-globin chains in response to fluctuating iron levels [2]. HRI is thought to exist as a homodimer in cells, bound to inhibitory heme molecules. When intracellular heme levels drop, these inhibitory heme molecules dissociate, leading to HRI activation [3-5]. Once activated, HRI undergoes autophosphorylation and subsequently phosphorylates its substrate – the alpha subunit of the eukaryotic initiation factor 2 (eIF2α) on serine 51 – resulting in ISR activation and up-regulation of the transcription factor ATF4.

More recently, HRI activity has been implicated in the regulation of mitochondrial stress and mitophagy, the autophagic elimination of mitochondria [6-10]. This process is primarily mediated through the signalling factor DELE1 (DAP-3 binding cell death enhancer 1). Under mitochondrial stress, the inner mitochondrial membrane protease OMA1 is activated and cleaves DELE1. The resulting C-terminal fragment oligomerises and accumulates in the cytosol, where it activates HRI and initiates the ISR. Furthermore, HRI activity may influence PINK1 kinase, mutations of which are associated with Parkinson’s disease [11]. However, it remains unclear how DELE1 stimulates HRI and whether this activation depends on heme release.

Beyond mitophagy, HRI also plays a role in broader proteostasis. It can be activated upon proteasomal inhibition (e.g., via compounds such as bortezomib) to facilitate proteasome-independent protein degradation via the lysosome. This mechanism contributes to chemotherapeutic resistance [12-14], though the precise means by which HRI is activated in this context remains to be elucidated.

Modulating HRI activity may have therapeutic benefits in cancer [13,15-18], as ISR overactivation can induce highly selective CHOP-mediated apoptosis in cancer cells. This has driven the development of HRI-activating compounds, such as 1-((1,4-trans)-4-aryloxycyclohexyl)-3-arylureas (cHAUs) and N,N′-diarylureas (e.g., 1-(benzo[d][1,2,3]thiadiazol-6-yl)-3-(3,4-dichlorophenyl)urea [BTdCPU]). However, the exact mechanism by which these compounds activate HRI remains elusive, with most studies being conducted in a cellular or cell lysate context [15,19-22]. Conversely, inhibiting the ISR and HRI in certain cancers, where constitutive pathway activation promotes cancer cell survival, may also have therapeutic potential [13,15,16,23,24].

Given HRI’s role in translation, cancer, and neurodegeneration, we have investigated human HRI and characterised it biophysically and biochemically. First, we mapped the autophosphorylation pattern of human HRI, identifying several novel phosphorylation sites, including tyrosine phosphorylation sites. We demonstrated that HRI autophosphorylation is inhibited by the clinically approved B-Raf inhibitors dabrafenib and encorafenib but not by LY3009120. Additionally, we found that GCN2iB, a tool compound used to selectively inhibit the related eIF2α kinase GCN2 [25], also inhibits HRI. However, we did not observe any direct *in vitro* effect of the small-molecule activator BTdCPU – despite its well-characterised activation of HRI in cells – suggesting that BTdCPU activates HRI indirectly.

Using hydrogen–deuterium exchange mass spectrometry (HDX-MS), we characterised the inhibitory mechanisms of both the heme analogue hemin and the B-Raf inhibitor dabrafenib. These two inhibitors show marked differences in their mechanism of action, suggesting very different modes of inhibition. Our data suggest that heme binding induces large-scale rearrangements within the protein, likely involving additional folding events of disordered segments, while dabrafenib binding resulted in a typical ATP-competitive inhibitor solvent exchange profile, with reductions in solvent exchange almost entirely localised within the kinase domain. These findings may provide insights into the future development of HRI inhibitors.

## Results

### HRI Oligomerisation state is phosphorylation independent

**Figure 1:**
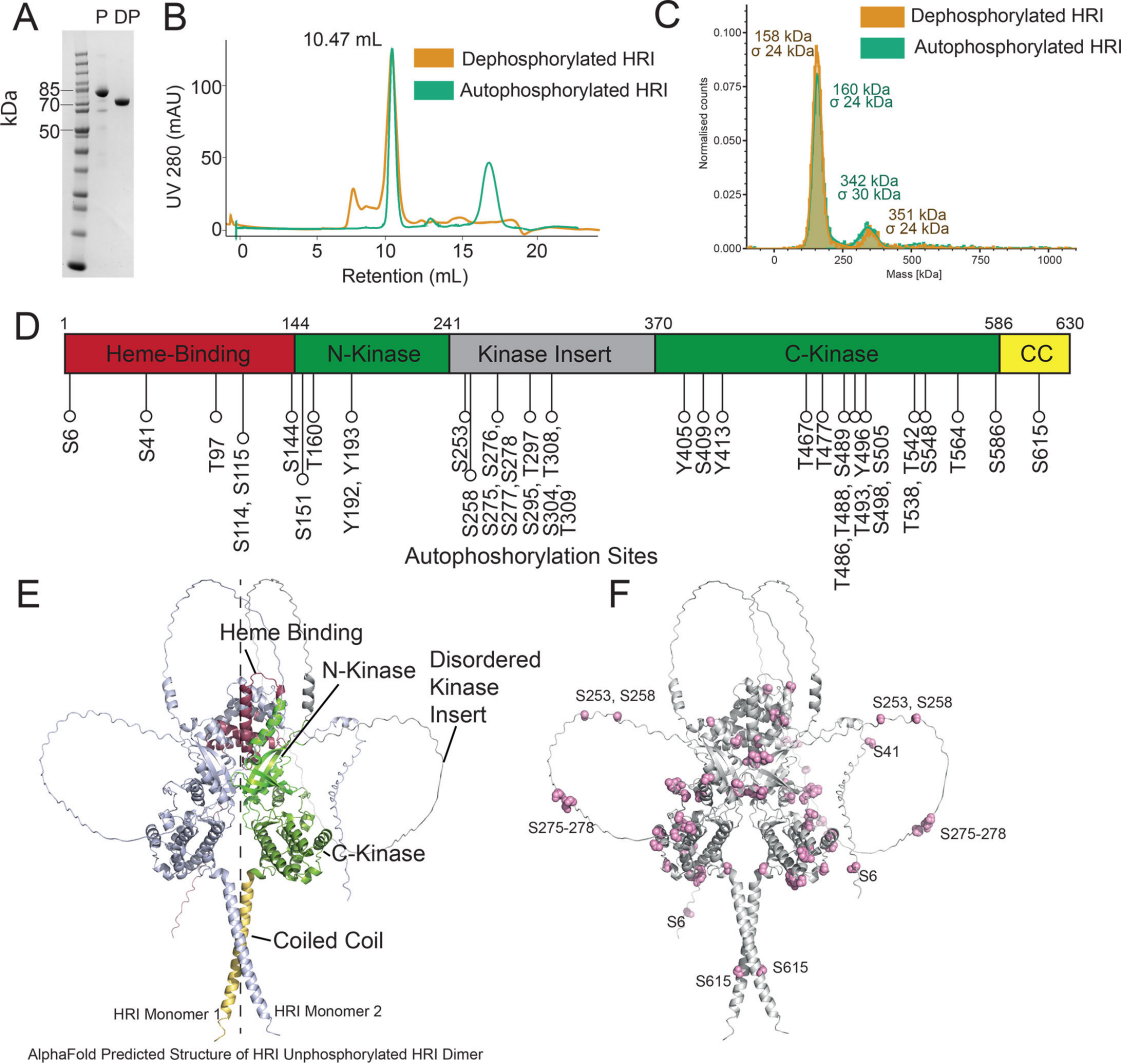
Characterisation of the phosphorylated HRI dimer. *(***A**) Purified recombinant HRI run on an SDS-PAGE gel in both autophosphorylated (P) and lambda protein phosphatase dephosphorylated (DP) protein. (**B**) Size-exclusion chromatography of autohosphorylated and dephosphorylated HRI both of which elute with a retention volume of 10.47 ml. (**C**) Mass photometry analysis of HRI. Both phosphorylated and dephosphorylated HRI have a predominant population of ~150 kDa, indicative of a dimeric oligomerization state. (**D**) Domain organization of HRI with sites of HRI autophosphorylation determined in this study. A single polypeptide of HRI contains a single kinase domain (split into the N-kinase and C-kinase sections) separated by a disordered kinase insert. CC = Coiled coil (**E**) AlphaFold 3 model of dephosphorylated HRI dimer, with domains highlighted. The dashed line shows the approximate line of 2-fold symmetry. (**F**) Locations of autophosphorylated residues on HRI.

N-terminally His-tagged human HRI was purified from *Escherichia coli* cells using affinity chromatography, ion exchange chromatography, and subsequent gel filtration (Figure 1) [4]. Gel filtration suggested the protein was homodimeric with an approximate mass of ~200 kDa (Supplementary Figure S1). SDS-PAGE analysis of the material purified from *E. coli* demonstrated HRI (monomeric molecular weight: 75017 Da) was running to approximately 85 kDa, suggesting post-translational modification. We treated HRI with lambda protein phosphatase until no phosphorylation modifications could be detected using mass spectrometry. Dephosphorylated HRI migrated further on the SDS-PAGE to ~75 kDa [4,19] (Figure 1A), while size exclusion chromatography also showed no difference in elution volume (Figure 1B, Supplementary Figure S1A and B). We conducted mass photometry analysis on dephosphorylated and autophosphorylated forms of HRI to determine whether phosphorylation caused any changes in the oligomeric state of HRI (Figure 1C). In both cases, we observed two peaks: a predominant peak with an average mass of approximately 150 kDa, indicative of a dimer, and a second minor peak of a possible quaternary state (with a mass of ~350 kDa). There was little difference in the relative abundances of these peaks for either the dephosphorylated and phosphorylated HRI, suggesting autophosphorylation does not drive dimerisation/oligomerisation.

### HRI undergoes extensive autophosphorylation on serine, threonine, and tyrosine residues

To investigate the locations of HRI autophosphorylations, we first dephosphorylated HRI using lambda protein phosphorylation (Supplementary S1C and D), removed the lambda phosphatase by gel filtration and then incubated this dephosphorylated HRI with ATP to induce autophosphorylation. Autophosphorylated HRI was then analysed by mass spectrometry to determine sites of phosphorylation. In total, 41 sites of autophosphorylation were detected (Figure 1D/F, Supplementary Data 1, Table 1), including the phosphorylation of T486 and T488 – two residues found within the activation loop and previously identified in mice (Mouse residues T483 and T485) as critical for autophosphoryation activity [27]. Unexpectedly, we also detected numerous phosphotyrosine residues. No phosphorylation sites were detected on a purified kinase-dead HRI mutant, HRI^K196M^ (Supplementary Figure S1).

**Table 1:** Autophosphorylation sites detected in human HRI.

Position	Peptide sequence	P-Site	*m/z*	z	M_r_ (experimental)	M_r_ (calculated)	Ion score	ID’d Prior?
-24–10^2^	MAHHHHHHSSGSENLYFQGSHMEFMQGGNSGVRK	S6	1344.4661	3	4030.3764	4030.3719	35	Yes [26]
11–52	REEEGDGAGAVAAPPAIDFPAEGPDPEYDESDVPAEIQVLKE	S41	1044.4710	4	4173.8551	4173.8467	49	Yes [26]
92–118	KLLCQTFIKMGLLSSFTCSDEFSSLRL	T97	1532.1959	2	3062.3773	3061.4000	61	Yes [26]
100–123	KMGLLSSFTCSDEFSSLRLHHNRA	S114 S115	919.0630	3	2754.1671	2753.1492	63	
134–146	RVRQDPCEDISRI	S144	727.8105	2	1453.6065	1453.6021	58	Yes [26]^1^
150–163	RSREVALEAQTSRY	S151	476.2280	3	1425.6623	1425.6613	51	
150–163	RSREVALEAQTSRY	T160	476.2286	3	1425.6618	1425.6613	68	Yes [26]
187–198	KLDGQYYAIKKI	Y192	639.8118	2	1277.6091	1277.6057	61	Yes [26]
187–198	KLDGQYYAIKKI	Y193	639.8109	2	1277.6073	1277.6057	52	Yes [26]
256–265	RAAIELPSLEVLSDQEEDRE	S253 S258	1087.4687	2	2172.9229	2172.9228	50	Yes [26]
256–265	RAAIELPSLEVLSDQEEDRE	S258	1047.4882	2	2092.9618	2092.9565	82	Yes [26]
270–289	KNDESSSSSIIFAEPTPEKE	S275	1009.4376	2	2016.8606	2016.8565	106	Yes [26]
270–289	KNDESSSSSIIFAEPTPEKE	S276 S277 S278	1089.4030	2	2176.7914	2176.7891	58	Yes [26]
291–307	RFGESDTENQNNKSVKY	S295 T297 S304	968.8440	2	1935.6735	1935.6690	54	Yes [26]^1^
303–315	KSVKYTTNLVIRE	T308 T309	485.2330	3	1452.6771	1452.6779	34	
401–422	RGREYVDESACPYVMANVATKI	Y405	1171.0101	2	2340.0057	2338.9963	66	
401–422	RGREYVDESACPYVMANVATKI	S409	780.6738	3	2338.9999	2338.9963	95	
401–421	RGREYVDESACPYVMANVATK	Y405 S409	864.6954	3	2591.0644	2591.0587	54	
401–422	RGREYVDESACPYVMANVATKI	Y413	780.6747	3	2339.0021	2338.9963	48	
458–473	KIGDFGLACTDILQKN	T467	815.8801	2	1629.7457	1629.7474	70	
458–480	KIGDFGLACTDILQKNTDWTNRN	T477	840.3826	3	2518.1261	2518.1199	71	
481–511	KRTPTHTSRVGTCLYASPEQLEGSEYDAKS	T486 T488 S489 T493	1158.4593	3	3472.3561	3472.3486	49	Yes [27]
481–511	KRTPTHTSRVGTCLYASPEQLEGSEYDAKS	S489 T493 Y496 S498	1158.7914	3	3473.3523	3473.3326	36	
490–511	RVGTCLYASPEQLEGSEYDAKS	S505	766.3301	3	2295.9686	2295.9606	40	
533–551	RAEVLTGLRTGQLPESLRK	T538 T542 S548	694.3163	3	2079.9270	2078.9204	32	Yes [26] ^1^
558–566	KYIQHLTRR	T564	505.7444	2	1009.4743	1009.4746	31	
571–597	RPSAIQLLQSELFQNSGNVNLTLQMKI	S586	957.1422	2	2868.4046	2868.4092	82	
609–622	KQLNLLSQDKGVRD	S615	725.8770	2	1449.7394	1449.7341	56	Yes [26]

1 Detected in mouse HRI

2 Identified in peptides containing the N-terminal His-tag

To better interpret the locations of these phosphorylated residues on HRI, we used AlphaFold 3 to create a prediction of the structure of dimeric human HRI [28] (Figure 1E and F) (Supplementary Figure S2). This prediction placed the kinase domains in a back-to-back conformation, with heme-binding domains in similar positions ‘on top’ of the kinase domain, with the coiled-coil domain situated beneath. A back-to-back conformation for the kinase domains is expected for the eIF2α kinases such as PKR and GCN2 [29,30]. However, due to large stretches of disordered residues (1–61 and 240–373), the overall prediction metrics were classed as ‘mediocre’ (ipTM = 0.49). Removing these sections improved these scores (ipTM 0.59).

### HRI can be inhibited by RAFi compounds and GCN2iB

**Figure 2:**
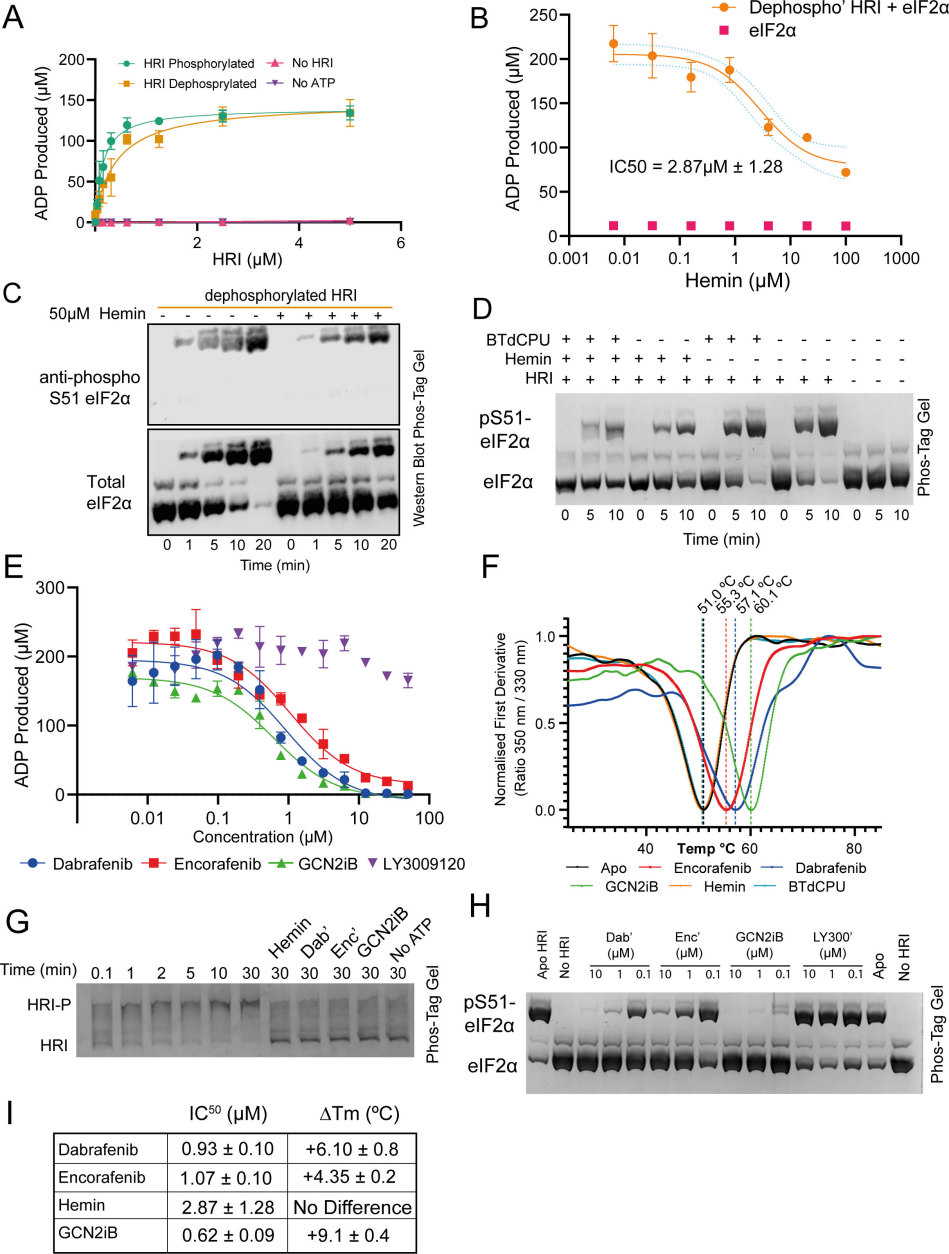
Characterisation of HRI modulators. *(***A**) ADP-GLO kinase assay of phosphorylated and dephosphorylated HRI against a recombinant human eIF2α substrate. Data are presented as the mean ± SD based on three independent experiments. (**B**) Concentration gradient of hemin inhibition using dephosphorylated HRI and recombinant human eIF2α as a substrate – an IC_50_ of 2.87 ± 1.28 µM was determined. No activity was detected in the absence of HRI (eIF2α only control). Data are presented as the mean ± SD based on three independent experiments. (**C**) Western blot assay from a Phos-Tag separated kinase assay using eIF2α substrate with dephosphorylated HRI. The Phos-tag gel was transferred to a membrane and probed with either a total eIF2α probe or a phosphoserine51-eIF2α probe to show the production of phosphorylated eIF2α over the course of the reaction, reduced in the presence of hemin. The pSer51 specific antibody probe shows the gradual production of phosphorylated substrate from HRI, which migrates slower through the Phos-tag gel. Blot representative of a triplicate experiment. (**D**) Phos-tag analysis of the effects of 10 μM hemin and/or 10 μM BTdCPU on eIF2α phosphorylation. BTdCPU did not appear to alleviate hemin inhibition. (**E**) ADP-GLO analysis of dephosphorylated HRI against various RAFi and GCN2iB. Data are presented as the mean ± SD based on three independent experiments. (**F**) nanoDSF analysis of 5 μM hemin, 10 μM BTdCPU, 10 μM dabrafenib, 10 μM encorafenib and 10 μM GCN2iB. T_M_s (as determined as the nadir of the first derivative of the ratio of the 350/ 330 nm curve) are shown above. (**G**) Phos-tag analysis of RAFi, hemin, and GCN2iB on HRI autophosphorylation. (**H**) Inhibition of HRI eIF2α phosphorylation by RAFis and GCN2iB (**I**) Table summarizing IC^50^ and *T*_m_ values of inhibitory compounds. *T*_m_ values are means ± SD based on three independent experiments.

To investigate how different inhibitors affect autophosphorylation and substrate phosphorylation, we next conducted inhibitor studies to discern their relative impact on these two processes. Previous studies have used the heme analogue hemin to investigate how iron levels affect HRI activity [3]. Using ADP-GLO kinase assays with a recombinant human eIF2α substrate, we determined that hemin inhibited HRI with an IC_50_ of 2.87 µM±1.28, which broadly agrees with previous studies which used an eIF2α-derived peptide as a substrate [3,31] (Figure 2A and B). We were unable to achieve ‘complete’ inhibition of HRI (as measured by ADP production) using hemin, with a residual ~25% ATPase activity detected even at high hemin concentrations ( > 200 μM) (Figure 2B). Phos-tag gel analysis indicated that hemin functionally blocked autophosphorylation (Figure 2G), but activity assays showed that there was still residual phosphorylation of eIF2α even at saturating concentration (Figure 2C and D). We then applied nano-differential scanning fluorimetry (nanoDSF) to determine the effects of compound binding on HRI stability. We observed an HRI unfolding event with a *T*_m_ of 51.0 ± 0.1°C and the addition of 5 μM hemin caused no significant difference to protein stability (50.7 ± 0.3°C).

Recently, it has been reported that small molecule BRAF^V600E^ inhibitors may have concentration-dependent inhibition/paradoxical activation against the related eIF2α kinase general control nonderepressible 2 (GCN2) [32,33]. Given the sequence homology between the kinase domains of HRI and GCN2 (Supplementary Figure S3)**,** we screened three BRAF^V600E^ compounds using the ADP-GLO kinase assay: dabrafenib, encorafenib, and LY3009120, and the tool compound and small molecule activator/inhibitor of GCN2, GCN2iB [25,34] (Figure 2E, F, H and I). We found that two of the RAFi compounds, dabrafenib (IC_50_ 930 nM±102) and encorafenib (1070 nM±104), were inhibitors of HRI, while LY3009120 had no effect on HRI activity. GCN2iB was a slightly more potent inhibitor of HRI with an IC_50_ value of 620 nM±88. Similar results were observed using the Phos-tag gels to monitor eIF2α phosphorylation. Thermal stability analysis (Figure 2F and I) showed significantly increased thermal stability to HRI on the addition of 10 μM these compounds – encorafenib increased the *T*_m_ by +4.35 ± 0.2°C, dabrafenib + 6.1 ± 0.8°C, and GCN2iB + 9.1 ± 0.4°C.

We also investigated the small-molecule BTdCPU – a well-characterised activator of HRI [16,19-22,35]. Despite a wide range of concentrations and approaches being employed, we did not detect any change in HRI activity upon BTdCPU incubation (Figure 2D, Supplementary Figure S4). We found that BTdCPU could not overcome hemin-induced inhibition and that in the absence of hemin did not activate HRI further. Addition of 10 μM BTdCPU caused no significant difference on HRI thermostability using nanoDSF analysis (50.8 ± 0.5°C) (Figure 2F).

### Hemin and dabrafenib binding result in distinct modes of HRI inhibition

**Figure 3:**
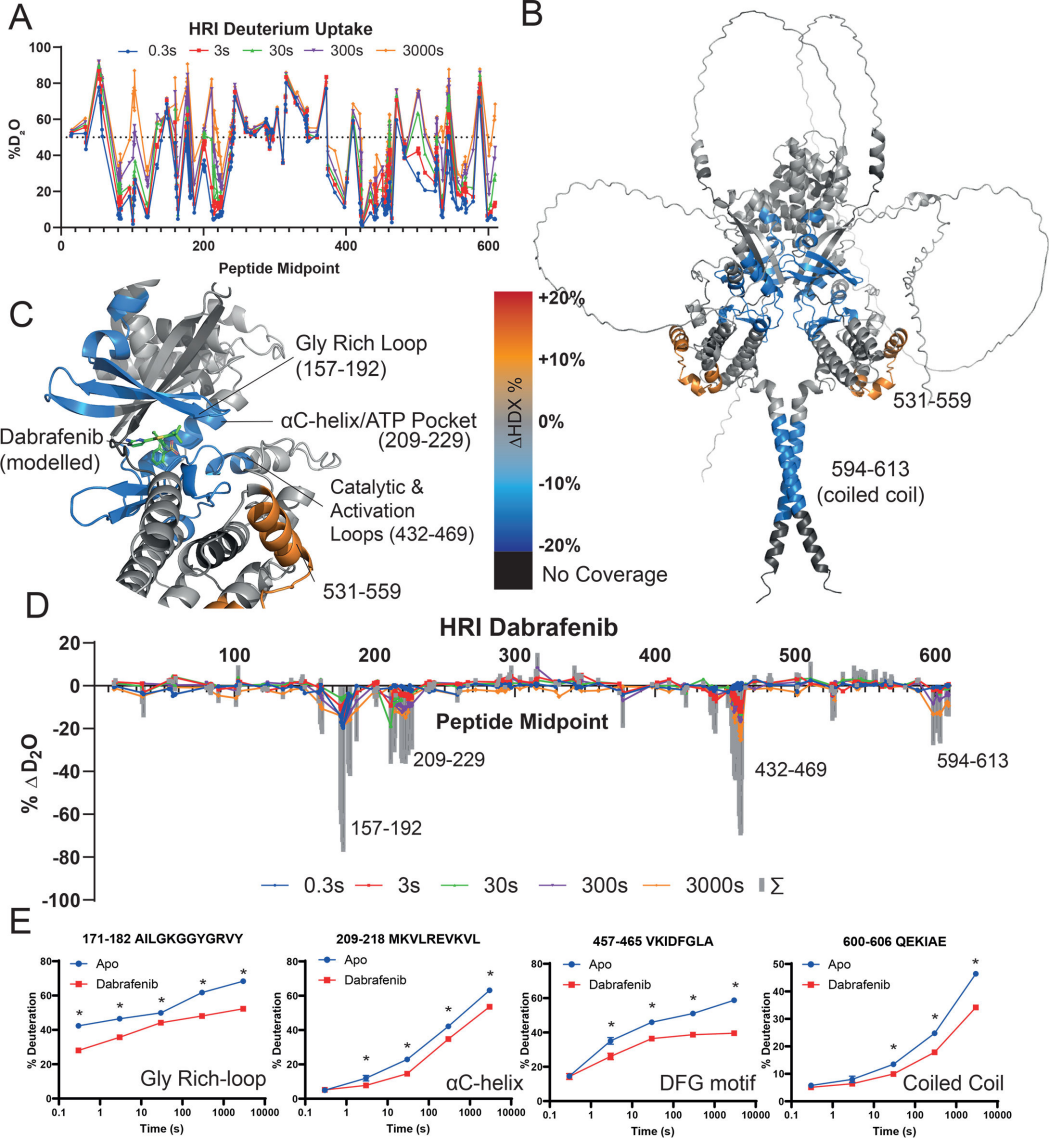
HDX-MS analysis of dabrafenib binding. *(***A**) Global Exchange profile of Apo Dimeric dephosphorylated HRI over five timepoints. The dashed line at 50% – typically peptides with >50% exchange at the 0.3 s timepoint (blue points) are disordered regions. (**B**) HRI kinase domain with dabrafenib binding modelled. Peptides which exhibited a reduction of solvent exchange upon dabrafenib binding are highlighted in blue, those with an increase in orange, and peptides with no coverage shown in black. Grey regions contain peptides which exhibited no significant change (i.e. between 5% and −5%). (**C**) Inset of the kinase domain of HRI, showing solvent exchange changes upon dabrafenib binding. Dabrafenib docked using a BRAF model (PDB:5CSW). (**D**) HDX-MS plot of dabrafenib binding to HRI. Summed difference over all five timepoints are shown as grey bars with individual timepoints show as points. (**E**) Selected peptides exhibiting differences in solvent uptake upon dabrafenib binding. Each point is a mean value with error bars of ± SEM. Many error bars are smaller than the point. Each exchange reaction was conducted independently in triplicate. * = > 5% and >0.5 Da difference and passes a student t-test with p = 0.05 (or lower).

To gain insight into the mechanism of these inhibitors, we conducted HDX-MS on dephosphorylated HRI in the absence or presence of 100 μM dabrafenib/hemin. Heme binding probably maintains HRI in an autoinhibited state, and thus, the biological context of heme binding would be prior to any activation event where phosphorylation may occur. We produced a peptide map of dephosphorylated human HRI consisting of 218 peptides, with a coverage of 89.3%, and average redundancy of 14.1. HDX-MS of Apo HRI identified that there were several unstructured loops, as determined by high rates of exchange (>50% at *t* = 0.3 s), maintained in dimeric HRI between residues 1–70, 143–153, 230–370, 467–495, and 583–593 (Figure 3A), which agreed well with the AlphaFold predicted structure (Table 2).

**Table 2:** HDX-MS statistics.

	Apo	Dabrafenib	Hemin
# of Peptides	218	218	218
Coverage	89.3%	89.3%	87.9%
Gaps of coverage	41–46, 66–72, 96–98, 106–113,366-389, 381–392, 516–521, 615–630		
Av. length	14.1	14.1	14.1
Redundancy	4.7	4.7	5.1
Time points	0.3 s^1^, 3 s, 30 s^2^, 300 s^2^, 3000 s^2^		
Replicates	ND 4, Deuterated 3		
Controls	Maximally deuterated (APO) Control		
Replicability^3^	1.08%	0.94%	~1.2%^4^
Significance threshold^5^	> 5%/0/.5 Da/Passes t-test		

1 0.3 s timepoint created by pipetting 3 s by hand with ice-cold solutions.

2 Conducted by liquid handling robot.

3 Mean standard deviation between replicates

4 Excluding EX1 peptides.

5 A peptide must meet all these criteria in order to have a significant change in solvent exchange.

Dabrafenib binding (Figure 3B–E) caused a reduction in solvent exchange largely concentrated around a kinase inhibitor pocket consisting of residues 157–192 (Gly rich loop), 209–229 (αC-helix), 432–469 (catalytic loop/DFG motif) (Figure 3B, C, D and E). Single peptides covering the DFG loop, for example, residues 459–464, showed strong reduction in HDX. However, neighbouring peptides (e.g., 448–456 and 467–475) showed no reduction, suggesting a possible direct interaction between dabrafenib and the DFG loop. Modelling of dabrafenib in the HRI kinase domain used crystal structure PDB:5CSW [36]. Unexpectedly, dabrafenib binding also produced an area of increased solvent exchange in the kinase C-lobe kinase domain between residues 531–559, and a reduction in solvent exchange in the coiled-coil region C-terminus residues 594–613 –sites predicted to be distant from the kinase domain catalytic site.

**Figure 4:**
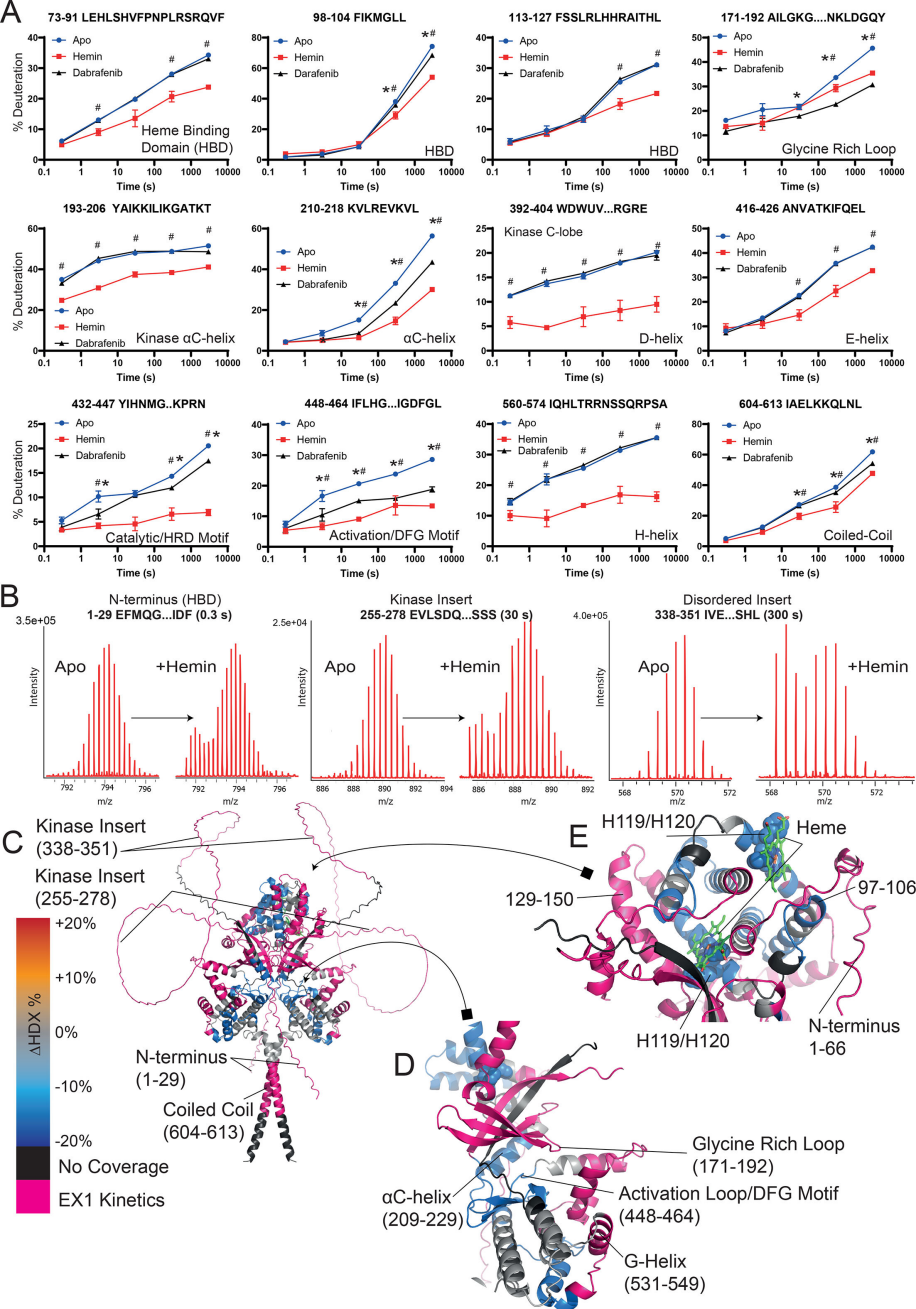
HDX-MS analysis of hemin binding. *(***A**) A selection of peptides detailing the solvent uptake differences upon hemin and dabrafenib binding. Each point is a mean value with error bars of±SEM. Many error bars are smaller than the point. Each exchange reaction was conducted independently in triplicate. * = > 5% and 0.5 Da difference and passes a student t-test with *P*=0.05 (or lower) with dabrafenib binding when compared with Apo HRI. # = > 5% and 0.5 Da difference and passes a student t-test with *P*=0.05 (or lower) with hemin binding when compared with Apo HRI. (**B**) Spectra of selected peptides exhibiting EX1 kinetics which emerge on hemin binding. (**C**) HDX-MS data mapped onto an AlphaFold Model with Heme docking. Peptides which exhibited a reduction of solvent exchange upon hemin binding are highlighted in blue, those with an increase in orange, and peptides with no coverage shown in black. Grey regions contain peptides which exhibited no significant change (i.e., between 5% and −5%). Peptides exhibiting EX1 kinetics are indicated as pink. (**D**) Kinase domain upon hemin binding highlighting catalytic and regulatory regions. (**E**) HDX-MS data around the Heme Binding Domain. Two previously identified residues, Histidine 119 and 120, are highlighted and represented as spheres. Heme molecules are shown in green, modelled using AlphaFold.

Contrastingly, hemin binding resulted in much more widespread alterations in the solvent exchange rate of HRI (Figure 4). First, residues 113–127 in the heme binding domain, which contain the heme binding histidine residues H119 and H120 [37], exhibited reductions in solvent exchange upon hemin-binding - regions of solvent protection not observed with dabrafenib binding. Furthermore, unlike dabrafenib binding, many HRI peptides exhibited bimodal spectral distributions upon hemin binding (Figure 4B, C and D). Bimodal populations are characteristic spectral patterns which can result from structural heterogeneity, or from ‘EX1’ solvent exchange kinetics (where the rate of protein opening/closing (*k_cl_*) is much slower than the solvent exchange rate (*k_ch_*)) [38,39]. Such EX1 kinetics arise when a continuous stretch of amino acids rapidly changes solvent exchange rate simultaneously, as may occur upon an induced folding event. Many HRI peptides also exhibited ‘classical’ EX2 exchange kinetics on hemin binding with prototypical isotopic distributions which also exhibited decreases – suggesting that incomplete hemin binding was not the root cause of the bimodal peptide populations. Within the kinase domain, for example, there is a large reduction in solvent exchange around the activation loop (the ‘DFG’ residues of 461–463) upon hemin binding. Another potential source of bimodal populations – peptide carryover – was also excluded as a possibility, with blank runs being conducted between samples and inspected for peptides from the previous run. Typical carry-over peptide intensity was less than 1% the previous run, suggesting that this was not the source of the bimodal peptide distribution.

Several regions of HRI predicted to be disordered and showing high exchange rates in the apo structure (typical of disordered segments) exhibited EX1 kinetics on hemin binding. The N-terminus of HRI exhibited EX1 kinetics between residues 1–70 on hemin binding (Figure 4B) – the N-terminus of HRI has previously been identified as undergoing a structural reorganisation upon heme binding [3,37]. EX1 kinetics were also observed in the disordered region of 144–153 (linking the heme-binding domain and N-lobe of the kinase domain), suggesting the possibility of a partial folding event occurring on hemin binding. Furthermore, within the N-lobe of the kinase domain, residues 171–208 also exhibited EX1 kinetics, as well as the very C-terminus of HRI (residues 596–613) addition of hemin, suggesting a widespread conformational change upon hemin binding. The large, disordered insert region of HRI found between the kinase lobes, residues 230 and 350, also demonstrated EX1 kinetics, with subpopulations of highly protected peptides emerging on HRI binding.

## Discussion

Here we present structural insight into the activation and inhibition of HRI via biochemical analysis and structural mass spectrometry, providing insights into how heme-like compounds, ATP-mimetic inhibitors and phosphorylation may work together to regulate this kinase. Before this study, it was unclear how the heme-binding domains of HRI could facilitate the inhibition of HRI. Here we show that HRI may undergo a large intramolecular rearrangement or folding event on heme binding, which ultimately prevents the kinase domain from autophosphorylating.

Both sequence alignment (online supplementary figure 3) and the AlphaFold predicted structure of HRI (Figure 1E) illustrate the kinase’s unusual architecture. The eIF2α kinase family is dimeric, most likely with a back-to-back architecture, although this interface is likely dynamic with large rearrangements upon activation [30], although currently no full-length dimeric eIF2α kinase structure has been empirically determined. Our HDX-MS data support the prediction that HRI has a prominent disordered insertion (residues 241–370) between the two lobes of the kinase domain, and a rather extended activating loop (residues 464 to 488). Furthermore, the P-loop (residues 170–181) lacks a consensus Walker Motif A (typically G-x [4]-GK-(TS)) [40] – HRI has a sequence of _166_EFEELAILGKGGY_178_ with only the GK residues adhering to the consensus sequence. Opposite the P-loop, the activation loop harbours multiple sites of phosphorylation, including T486 and T488, which have been shown to be crucial for activation of the kinase [27]. Currently, it is not clear how these loops alter HRI regulation and activity, although our HDX-MS data and phosphorylation mapping show these large loops have central roles in the activation of HRI.

HRI readily autophosphorylates, and 33 sites have been reported previously – although these studies were conducted in mice HRI [26]. Here in a recombinant setting, free of alternative kinases, we report a total of 41 autophosphorylation sites on human HRI. Although mouse and human HRI have ~83% sequence homology (online supplementary figure 5), some of these sites of phosphorylation are unique to either species. There are two notable patches of dense phosphorylation – a section in the disordered insert (residues 275–309), and within the kinase activation loop of HRI. While phosphorylation of the activation loop is a common means of controlling kinase activation, in HRI we observed the phosphorylation of T486, T488, S489, T493, Y496, and S498, six residues within the 12 amino acids, representing a dense patch of phosphorylation.

Interestingly, we detected numerous phosphotyrosine modifications: some have been detected previously [26], but the material purified in previous studies could not exclude the possibility of extraneous kinases modifying HRI. Of particular importance is the residue Y193 (mouse numbering), which was previously found to be critical for HRI activity [26] – a Y193F mutant had ~50% activity of wtHRI. Here, we identified the phosphorylation of both Y192 and Y193 (although in mice, Y192 is H192, preventing its phosphorylation). Our material, purified from *E. coli* and dephosphorylated with lambda phosphatase before subsequent autophosphorylation, leaves limited opportunity for these sites to be the result of extraneous phosphorylation. Furthermore, using a kinase-dead HRI mutant K196M, we did not detect any phosphorylation modifications on HRI.

An example of a protein kinase family that can both tyrosine autophosphorylate and phosphorylate substrates on serine/threonine residues is the DYRK (dual specificity tyrosine-phosphorylation regulated kinase) family. These proteins have been shown to tyrosine autophosphorylate co-translationally and after translation as a mature protein [41]. A key determinant of specificity for serine/threonine versus tyrosine kinases is a single amino acid in a region of the activation segment known as the *P*+1 loop [42]. In serine/threonine kinases, this residue is a serine or a threonine, and in tyrosine kinases, it is a proline essential for interacting with the phospho-acceptor tyrosine residue. In dual-specificity kinases, for example in the DYRKs, it is a serine [43], and in HRI T488 is followed by a serine (^481^GKRTPTHTSRV^491^), and several other features of the kinase domain point towards it being a Ser/Thr Kinase (e.g., the APE-6 ‘GT’, the HRD+2 ‘K’, and the lack of a double YY at the DFG+10 position [44]). Other elements point towards Tyr/Kinase, e.g., HRD+4 ‘RN’ [44]. Notably, the highly conserved ‘APE’ element is found as ApSPE in HRI. Comparing HRI to the DYRK 1A/2/3, many sequences and residues are conserved, namely the catalytic loop, e.g. a ‘HRDLKPxNIxL’ motif and a cysteine at the +2 position of the DFG (DFGxxC) – this cysteine is not present in the other eIF2α kinases (Supplementary Figure S3). Mutagenesis and further structural studies may help elucidate how the HRI kinase domain is capable of tyrosine phosphorylation. Although there is little evidence for substrates other than eIF2α for HRI (e.g., GCN2 has been linked to FBXO22 phosphorylation [45]), it remains to be seen if HRI can phosphorylate tyrosine residues of substrates other than itself.

Here, we report that the small molecule B-Raf inhibitors dabrafenib and encorafenib, as well as the GCN2 inhibitor GCN2iB, inhibit HRI *in vitro,* which may point to future drug development. Given that both B-Raf and HRI function as homodimeric back-to-back kinases, it may be of interest to determine whether these compounds also exhibit the same concentration-dependent paradoxical activation behaviour as observed in B-Raf [46]. We did not observe activation of HRI by these compounds, but further assay optimisation may be required to observe additional increase in activity. HDX-MS provides a clear indication of the binding site for dabrafenib within the kinase domain (Figure 3B and E), with multiple protections in the kinase site as would be expected for a kinase inhibitor. There are reductions in solvent exchange in residues corresponding to both the P-loop/Gly rich loop (residues 170–183), the catalytic loop (441-449), and DFG motif (461-463) and part of the activating loop/HRD motif (464-508). The two crucial autophosphorylation residues of T486 and T488 showed no reduction in solvent exchange upon dabrafenib binding, which may mean that these residues can still be phosphorylated and potentially ‘activate’ the enzyme while dabrafenib is still bound. Dabrafenib is a ‘type 1.5’ inhibitor for BRAF^V600E^, binding the active conformation of the kinase with the DFG loop ‘in’ and αC-helix out [36] – it is not clear from our data whether this mode of binding is maintained in HRI.

Additionally, dabrafenib binding caused an increase in the solvent exchange rate in the kinase domain in residues 531–559. The role of these residues isn’t readily apparent – the residues in the kinase domain may contain a cryptic Walker B motif, although there is considerable variability in this motif. This region does contain residues T538, T542, and S548, all of which are autophosphorylated by HRI. Dabrafenib binding also resulted in the protection in part of the coiled-coil domain in residues 594–613. Given the predicted distance between the kinase domain and this section of the coiled-coil, it is difficult to interpret this, although previous studies have highlighted the importance of this coil in maintaining the dimer of HRI – a decrease in solvent exchange may be the result of a tighter interaction between these helices, resulting in a more stable dimer [3] – also supported by the increased thermal stability we observed with the nanoDSF data.

Hemin appears to be an incomplete inhibitor of HRI – other inhibition assays show a similar profile of inhibition [3-5]. Here, we show that this incomplete inhibition manifests as hemin binding appearing capable of preventing widespread autophosphorylation of HRI while still allowing for some eIF2α phosphorylation (Figure 2D). Incomplete inhibition may be because hemin is patently not heme – in a cellular context, there will be a variety of hemes which have different chemistries with different functional groups. For example, hemin contains ferric (Fe^3+^) rather than ferrous (Fe^2+^) iron, as found in heme. It may well be that certain hemes are more potent inhibitors of HRI. However, we did find that hemin binding resulted in reductions in solvent exchange at H119/H120 – a proposed heme binding site of HRI [37]. A further conserved residue C411 (see Supplementary Figure S5) has been implicated in heme binding. Although this exact residue did not appear to have a reduction in solvent exchange, the neighbouring helices of 416–426 and 392–408 both had reductions in solvent exchange (Figure 4D). Given C411’s predicted distance from the heme binding site, this further supports that heme binding may result in widespread structural rearrangement.

Overall, previous mutagenesis studies had also suggested that heme binding causes large-scale structural rearrangements of HRI [26,27,37,47], and this had been accompanied by changes in helicity as measured by circular dichromatism. Recent truncation analysis by Ricketts *et al*. suggests that hemin may be directly binding to the kinase domain rather than the heme-binding domain of HRI [3], although the authors were careful to point out this may be a result of their truncation rather than the biologically relevant mechanism of heme inhibition. Our data suggest that heme may well be interacting with the kinase insert region of the kinase domain, resulting in the observed EX1 kinetics in this region.

Finally, BTdCPU has been widely used as an HRI activating compound [10,16,35] – although its mode of action was unclear – previous studies had suggested that N,N′-diarylureas were capable of directly binding and activating HRI [22]. We were unable to detect activation of HRI by BTdCPU under numerous differing conditions (Figure 2, Supplementary Figure S4). To facilitate HRI activation, the previously suggested model would require BTdCPU to displace a heme molecule to facilitate activation – this would require a *Kd* of <1 μM, but we were unable to detect any effect on HRI activity even at concentrations of greater than 100 μM. This agrees with a recently published study by Perea *et al.* who demonstrated that HRI activation via BTdCPU functioned via mitochondrial decoupling and subsequent OMA1-DELE1 signalling, rather than direct interaction with HRI [10].

Given the expanding role of HRI in mitochondrial stress sensing [6-10], it is likely that there will be renewed interest in targeting HRI in disease. Our data here show the BRAF^V600E^ compounds dabrafenib and encorafenib, along with GCN2iB, are inhibitors of HRI – however, there is the distinct possibility that these compounds may also activate HRI via paradoxical inhibitor activation mechanisms [25,48]. A recent study found evidence for activation of HRI via two nucleoside mimetics, suggesting that HRI may also be capable of paradoxical activation by ATP-like molecules [49]. Further structural work is necessary to understand HRI inhibition and activation, as current models fail to completely explain the structural changes observed with heme binding.

## Materials and methods

## Protein Purification

*HRI Expression and Purification:* Briefly Human HRI/HRI K196M (the kinase dead mutant) cDNA was obtained from the MRC Protein Phosphorylation and Ubiquitination Unit (MRC PPU) and was subcloned into a pOPTH plasmid with an N-terminal 6His TEV-cleavable tag. Protein was expressed in BL21-Rosetta(DE3) (Novagen) *E. coli*. Cells were grown at 37°C in 2xTY media until an absorbance of 0.7 at 600 nm was achieved, where protein expression was induced on the addition of 0.8 mM IPTG. Cells were left to express protein for 16 h at 18°C. A pellet generated from 3 L of media was lysed in 100 ml lysis buffer (20 mM Tris pH 8.0, 500 mM NaCl, 20 mM Imidazole, 2 mM beta-mercaptoethanol (BME), with 1 µl benzonase (Novagen) and 2 EDTA-free Protease inhibitor tablets (PROMEGA)) via probe sonication. Lysate was centrifuged at 4°C at 40,000 *g* for 45 minutes. The supernatant was filtered through a 0.45 nm filter and then loaded onto a 5 ml HisTrap HP Column (Cytiva) equilibrated in Lysis Buffer. After washing with 10x CV with lysis buffer and an mAU of <100 achieved, a gradient of NiNTA Buffer B (20 mM Tris pH 8.0, 500 mM NaCl, 200 mM Imidazole pH 8.0, 2 mM BME) was applied. Fractions were analysed using SDS-PAGE to determine HRI-containing fractions. HRI-containing fractions were then diluted 1:1 with Q_O_ Buffer (20 mM Tris pH 8.0, 2 mM BME), and then passed over a 5 ml Q HP (Cytiva) column, equilibrated in Q_A_ Buffer (20 mM Tris pH 8.0, 100 mM NaCl, 2 mM BME), at a rate of 2 ml/min. A gradient of increasing Q_B_ buffer (20 mM Tris pH 8.0, 1 M NaCl, 2 mM BME) was then initiated, with HRI eluting at approximately 600 mM NaCl. HRI-containing fractions were then concentrated using a VIVASPIN 10 k MWCO Concentrator (Sartorius) until a volume of <1 ml was produced. We then dephosphorylated HRI using Lambda Protein Phosphatase (Lambda PP (New England Biolabs)), with 800 units of phosphatase, and the reaction left at 4°C for 16 hours. Finally, dephosphorylated HRI was injected onto a Superdex 200i GL 10/30 (Cytiva) equilibrated in Gel Filtration Buffer (20 mM HEPES pH 7.5, 150 mM NaCl, 2 mM TCEP). HRI-containing fractions, eluting at ~10.5 ml, were pooled and concentrated with a VIVASPIN 10 k MWCO Concentrator (Sartorius) until a concentration of 1.5 mg/ml was achieved. HRI was then frozen in liquid nitrogen and stored at -70°C. Gel Filtration calibration was conducted using the Cytiva Gel Filtration HMW Calibration Kit (28403842) with the manufacturer’s suggested mix of proteins for the Superdex 200 10/30 column, using the Gel Filtration Buffer as described above.

*eIF2α Expression and Purification:* Expression and purification of recombinant human eIF2α was conducted as described previously [50]. DNA encoding full-length human eIF2α (NCBI reference number: NP_004085.1) was inserted into the vector pOPTH with an N-terminal His6 tag followed by a TEV protease site. The plasmid was transformed into chemically competent BL21 Star (DE3) cells, and cells were grown overnight before being inoculated to a 50 ml starter culture in 2xTY media containing 0.1 mg/ml ampicillin. The starter culture was incubated at 37°C for 90 minutes, then 10 ml starter culture was added to 4 × 900 ml 2xTY media containing Ampicillin. Cultures were incubated at 37°C until the optical density reached 0.7, and then protein expression was induced by the addition of 0.3 mM isopropyl β-D-1- thiogalactopyranoside (IPTG). Cells were grown for a further 3 hours at 37°C before being harvested, washed with ice-cold phosphate-buffered saline and frozen in liquid nitrogen. Bacterial cell pellets were lysed in 100 ml lysis buffer (20 mM Tris-HCl pH 8.0, 100 mM NaCl, 5 % v/v glycerol, 2 mM BME, 0.5 mg/ml lysozyme (Sigma L6876), 2 U/ml benzonase, one complete EDTA-free protease inhibitor tablet (Roche 04693132001) per 50 ml of buffer). Cells were lysed using a probe sonicator for 5 minutes (10 s on/ 10 s off) and then centrifuged at 140,000 *g* for 45 min at 4°C. The supernatant was filtered through a 0.2 µm syringe filter before being loaded onto a 5 ml HisTrap HP Column (Cytiva 17524801) equilibrated in Ni A Buffer (20 mM Tris pH 8.0, 100 mM NaCl, 5 % v/v glycerol, 10 mM imidazole pH 8.0, 2 mM BME), followed by the elution of protein via a gradient of Ni B Buffer (20 mM Tris pH 8.0, 100 mM NaCl, 5% v/v glycerol, 200 mM Imidazole pH 8.0, 2 mM BME). Protein purification then proceeded as described for HRI. Proteins were concentrated to ~10 mg/ml and then snap frozen in liquid nitrogen.

### Phos-tag assays

250 nM phosphorylated or unphosphorylated HRI incubated with 8 µM eIF2α and 100 µM ATP in Kinase Reaction Buffer (20 mM HEPES pH 7.5, 150 mM NaCl, 5 mM MgCl_2_, 1 mM TCEP) for stated time at either room temperature or on ice. Hemin (Selleckchem S5645) was dissolved in 10 mM NaOH [4]. Reactions were terminated through the addition of an equal volume of 2 x Phos-tag Loading Buffer (0.1 M Tris pH 6.5, 0.2 M DTT, 4% w/v SDS, 15% Glycerol, 1 mM ZnCl_2_). Precast 17-well Phos-tag gels (SuperSep PhosTag (50 µmol/L) FujiFilm 192–18001/199-18011) were loaded with 10 µl of the reaction/Loading buffer solution in the suggested running buffer (0.1 M Tris Base, 0.1 M MOPS, 0.1% SDS-PAGE, 1 mM NaHSO_3_). Gels were run for 1 hour at 150 V and stained using Quick Coomassie Stain (Generon).

### Western blots

For Phos-tag Assays, gels were washed three times in transfer buffer (25 mM Tris, 190 mM Glycine, 10% Methanol, pH 8.3) with 10 mM EDTA. Proteins were transferred using wet transfer. All blocking buffers were 4% BSA in TBST. Antibodies used: HRI (Invitrogen 7H3L3, Rabbit, 1:500), Total eIF2α (Santa Cruz Biotechnology, SC-133132, 1:500), pSer51 eIF2α (Cell Signalling Technology 9721S, 1:250), IRDye680 Goat anti Mouse (Licor 926–68070), IRDye 800CW Donkey Anti Rabbit (Licor 926–32213). All images taken using the Licor Odyssey FC system and images analysed using ImageStudio. Blots representative of a minimum of three repeats.

### ADP-GLO kinase assays

Using a PROMEGA ADP-GLO Kinase assay, 100 nM HRI was incubated with 5 µM eIF2α and 100 µM ATP (or at the stated concentrations) in Kinase Reaction Buffer (20 mM HEPES pH 7.5, 150 mM NaCl, 5 mM MgCl_2_, 1 mM TCEP) for the stated time at room temperature, until the reaction was quenched using the ADP-GLO Reagent. 4 µl kinase reactions were conducted in Corning 3824 wells before quenching and development using the ADP-GLO/Kinase Detection Reagent as per the manufacturer’s instructions. An ADP/ATP curve was created using the kit for calculation of relative ADP concentrations after kinase reaction quenches. Luminescence measured using a Pherastar CLARIOstar (BMG LABTECH). Data analysis conducted using GraphPad Prism 10 (GraphPad). A simple linear regression model for the ADP calibration curve was used to determine the relationship between luminescence and ADP concentration. Non-linear regression analysis (typically Agonist vs. response (three parameters)) was used in hemin/BTdCPU/RAFi experiments.

### AlphaFold prediction

The human HRI sequence was used as a basis for AlphaFold 3(27), with 2 copies of unphosphorylated HRI being set as a parameter for dimerisation. Heme was also included for the prediction of heme binding sites.

### Mass photometry

Mass determination of solution phase HRI and phosphorylated HRI hemin was conducted using a Refeyn TwoMP instrument. Experimental data were collected using Grace Bio-Labs Culture Well Reusable 50–3mm DIA x 1 mm Depth Gaskets and High Precision glass microscope slides, repeatedly cleaned in ultrapure water and isopropanol and dried using a stream of nitrogen gas. Data were obtained through the collection of mass photometry videos. Prior to HRI data collection, mass calibration was conducted using BSA (66 kDa) and aldolase (160 kDa). By fitting Gaussian functions to ratiometric contrast values obtained from the protein standards using the DiscoverMP v2.5 software (Refeyn), a linear mass calibration was obtained. HRI proteins were diluted to the stated concentrations in 20 µl 20 mM HEPES pH 7.5, 150 mM NaCl, 5 mM MgCl_2_ and 2 mM TCEP, placed on the slide, and then a one-minute mass photometry video was collected. Data was analysed using the DiscoveryMP v2.5 software.

### Thermal unfolding assays

Thermal Unfolding Assays (nanoDSF) were conducted using a NanoTemper Panta using Prometheus Standard Capillaries (NanoTemper) (PR-C002). Briefly, HRI was defrosted and centrifuged at 20,000 g for 10 minutes, and then diluted to 0.2 mg/ml in 20 mM HEPES pH 7.5, 150 mM NaCl, 1 mM TCEP. HRI was incubated with hemin/BTdCPU/RAFi on ice for 45 minutes before being analysed. A gradient of 1 °C/min was applied to the sample starting from 25 °C to 85 °C. Fluorescence at 330 nm and 350 nm was monitored using an excitation wavelength of 280 nm. Data analysis and identification of inflection points/TMs was conducted using the Panta Analysis Software (NanoTemper).

### HRI autophosphorylation assay and mass spectrometry

Lambda phosphatase treated HRI was produced as detailed above. 1 mg/ml HRI was incubated with 200 µM ATP for 1 hour at room temperature, before being quenched with SDS-PAGE Loading buffer and running on a 4–12% BisTris Gel in MES buffer for 45 minutes. Bands corresponding to the gel-shifted HRI protein (there was no protein at the lower, non-phosphorylated starting migration) were excised from the gel and sent for mass spectrometry analysis. Briefly, gel bands were washed sequentially for 15 min at RT in ~200 µl of 1:1 MPW/Acetonitrile (ACN), 100 mM Ammonium Bicarbonate (Ambic), 1:1 Ambic/ACN, then a final wash in ACN before desiccation in SpeedVac for 10 min. Bands were then reduced in 10 mM DTT, 20 mM Ambic for 60 at 56°C, followed by a wash in 50 mM Indole-3-acetic acid, 20 mM Ambic for 30 min at RT. Bands were then washed in the Ambic, Ambin/ACN, and ACN for 15 min before drying again with the SpeedVac. Gels were then digested using Trypsin (Pierce) at 12.5 µg/ml, 20 mM Ambic, overnight at 30°C. Peptides were then extracted via incubation with ACN for 15 min. The gel was then centrifuged, and peptides recovered from the supernatant. To this solution, 5% formic acid was added and incubated for 45 minutes. Samples were dried down and then reconstituted with 10 µl 5% formic acid/ 10% ACN and then diluted with 40 µl of MPW. Solutions were then analysed by mass spectrometry using a Thermo QExactive Plus Orbitrap. Data was processed using Mascot. Only peptides with ppm scores < 5, Ion Scores > 30, and unambiguous MS/MS assignment of phosphorylation modification assignment were included in results. Experiments were conducted in triplicate, and only assignments found in all three repeats are included in the results.

### HDX-MS sample preparation

5 µM dephosphorylated HRI was incubated with or without 100 µM hemin/dabrafenib for 30 minutes in Protein Dilution Buffer (20 mM HEPES pH 7.5, 150 mM NaCl, 2 mM TCEP). 5 µl of this sample was diluted with 55 µL of Deuteration Buffer (20 mM HEPES pH 7.5, 150 mM NaCl, 2 mM TCEP, 1% DMSO, 96.5% D2O) (final D2O concentration = 85.95%) for timepoints 3/30/300 / 3000 s, before being quenched with 20 µl of ice-cold Quench Solution (6 M Urea, 2% Formic Acid), and being snap frozen in liquid nitrogen and stored at –70°C. A further time point, 0.3 s, was achieved by incubating both the sample and deuteration buffer on ice and then conducting a 3 s exchange reaction. Each exchange reaction was conducted independently four times. A maximally deuterated control where denatured HRI was incubated in 98% D_2_O for 24 h was also conducted to correct for back-exchange.

### HDX-MS data acquisition

Data acquisition was conducted broadly as previously described ([51]). Samples were rapidly thawed at room temperature and then injected into automated HDX-MS fluidics and UPLC manager system (Waters). Samples were loaded into a 50 µl loop and then subsequently digested using a Waters Enzymate BEH Pepsin Column (Part No. 186007233) in a 0.1% Formic Acid solution with a flow rate of 200 µl/min at 20°C. Peptic peptides then flowed onto a Waters ACQUITY UPLC BEH C18 VanGuard Pre-Column at 1°C (Part No. 186003975). After digestion, the flow path was changed to elute the peptides via a Waters ACQUITY UPLC BEH C18 1.7 µm 1.0 × 100 mm reverse phase column (Part No. 186002346). A gradient from 0-85% 0.1% Formic Acid/ Acetonitrile, conducted at 1°C with a 40 µl/min flowrate, was used to elute deuterated peptides which were then ionised using an ESI source. Mass spectrometry data were collected using a Waters Select Series cIMS instrument, from a 50–2,000 m/z range with the instrument in HDMSe mode. A single pass of the cyclic ion mobility separator (with a cycle time of 47 ms) was conducted. A blank sample of protein dilution buffer with quench was run between samples, and carryover was routinely checked to be <1% intensity of the prior sample.

### HDX-MS data analysis

Peptide sequence identification was conducted using Protein Lynx Global Server (Waters). Minimum inclusion criteria were a minimum intensity of 5000 counts, minimum sequence length 5, maximum sequence length 35, a minimum of three fragment ions, a minimum of 0.1 products per amino acid, a minimum score of 5.0, a maximum MH+Error of 10 ppm. Subsequent analysis and determination of deuteration values conducted using HDExaminer (Sierra Analytics/Trajan). Experimental design, data acquisition, analysis and reporting are in line with the community agreed recommendations [39].

## Supplementary material

Online supplementary figure 1

Online supplementary figure 2

Online supplementary figure 3

Online supplementary figure 4

Online supplementary figure 5

Online supplementary material

## Data Availability

All data and reagents are available from the authors upon request. Uncropped western blots, SDS-PAGE and Phos-Tag gels are included as Supplementary Data. The HRI autophosphorylation mass spectrometry data have been deposited to the ProteomeXchange Consortium via the PRIDE partner repository with the dataset identifier PXD064940 and 10.6019/PXD064940

## Abbreviations

GCN2: general control nonderepessible 2
HDX-MS: hydrogen–deuterium exchange mass spectrometry
HRI: heme-regulated inhibitor
ISR: integrated stress response
