# Supplementary material for: Structural insights into allosteric inhibition of HRI kinase by heme binding via HDX-MS
Source: Biochem J. 2025 Jun 17;482(12):859–75. doi: 10.1042/BCJ20253072 (PMC12235045; doi:10.1042/BCJ20253072)
Supplement: Online supplementary figure 1 [file bcj-482-12-BCJ20253072-supp1.pdf]

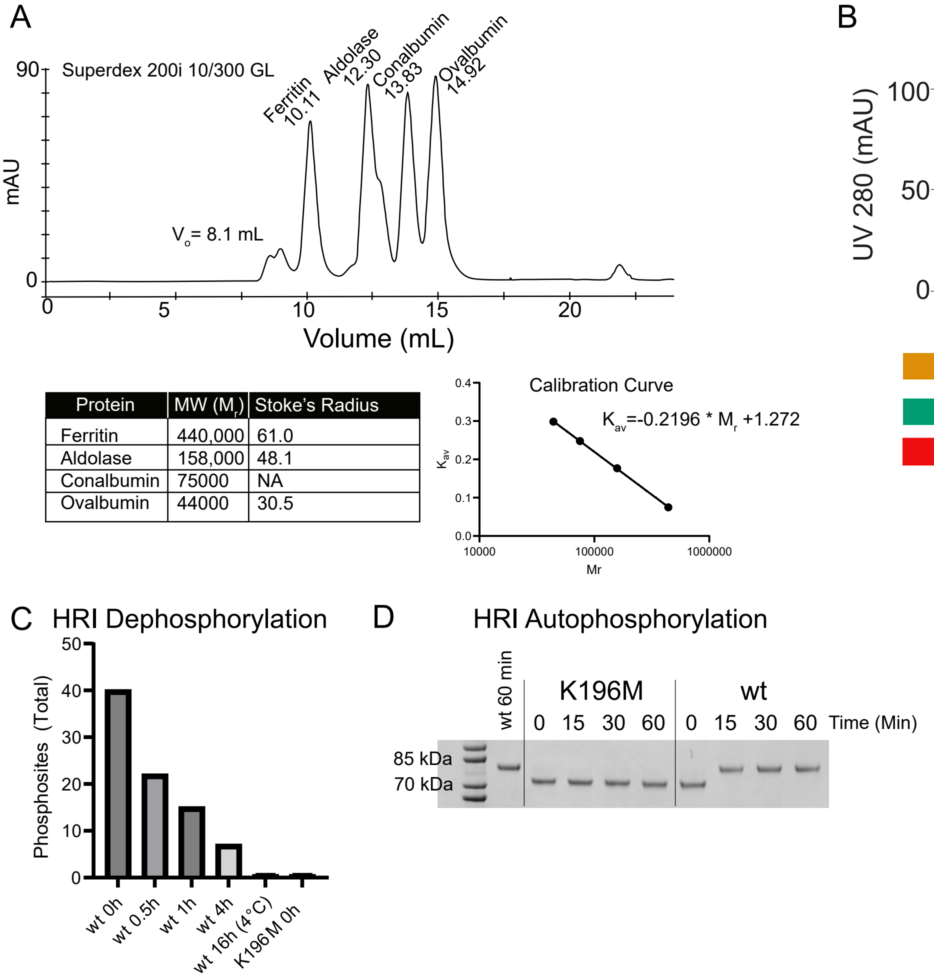

Supplementary Figure 1: Further Characterisation of Phosphorylated HRI, Dephosphorylated HRI, and HRI K196M. (A) Gel Filtration Profile and calibration curve for high molecular weight standards (Cytiva). (B) Gel Filtration Profile of dephosphorylated HRI, phosphorylated HRI, and HRI K196M. This is the same gel filtration curve as shown in Figure 1B. (C) Dephosphorylation timecourse of wtHRI and HRI K196M. HRI (purified from E.coli) was incubated with Lambda Protein phosphatase at either room temperature or at 4 °C for the stated times. Sites of autophosphorylation were identified by mass spectrometry. No autophosphorylation sites were observed on HRI K196M after purification. (D) HRI Autophosphorylation timecourse. Dephoshoylated HRI or HRI K196M was incubated with 200 μM ATP for the stated times.
