# Supplementary material for: Structural insights into allosteric inhibition of HRI kinase by heme binding via HDX-MS
Source: Biochem J. 2025 Jun 17;482(12):859–75. doi: 10.1042/BCJ20253072 (PMC12235045; doi:10.1042/BCJ20253072)
Supplement: Online supplementary figure 2 [file bcj-482-12-BCJ20253072-supp2.pdf]

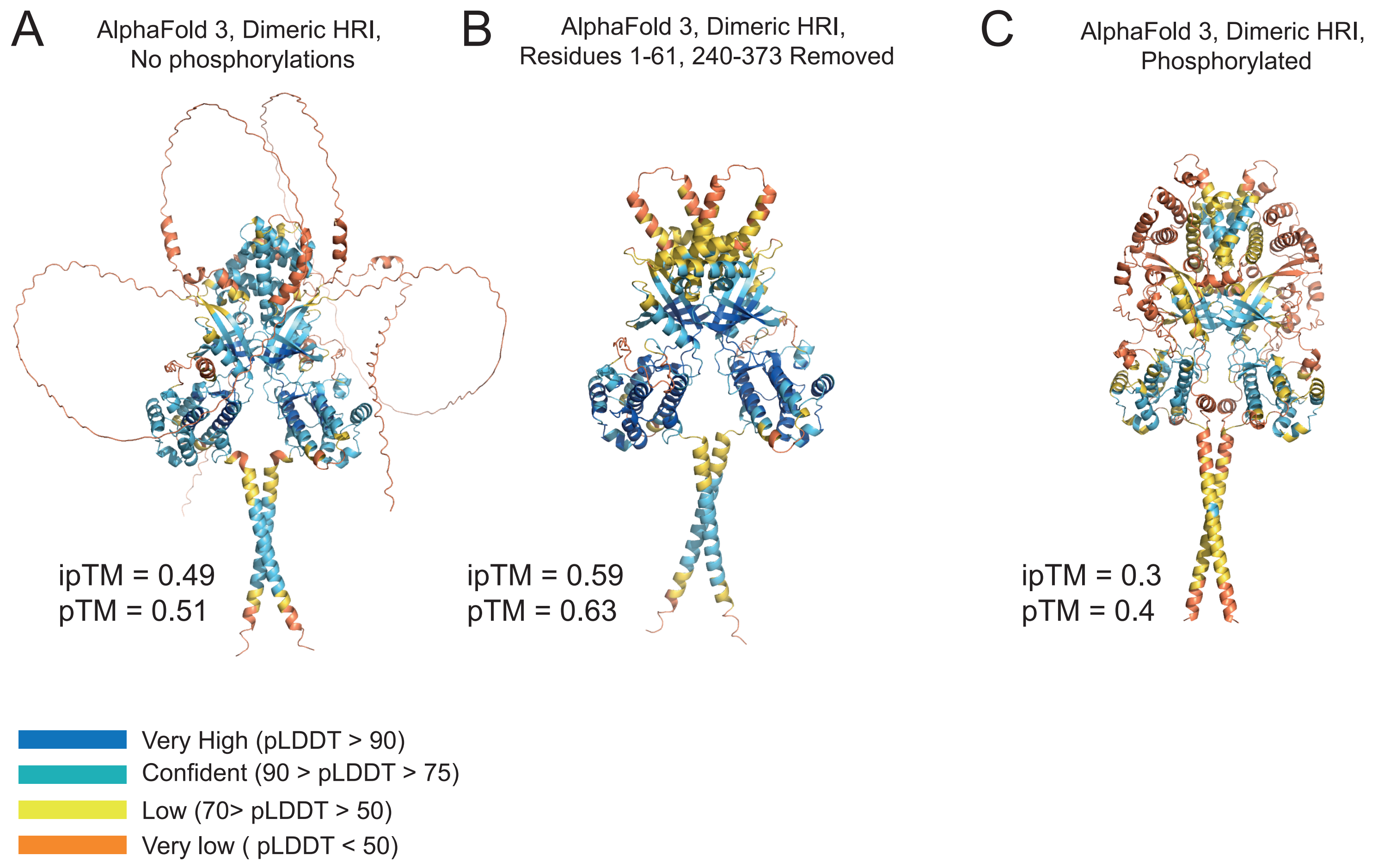

Supplementary Figure 2: AlphaFold 3 predictions of HRI. Predictions were generated using two copies of human HRI sequence as an input. (A) Full-length human HRI (B) A truncated version of HRI removing segments predicted to be disordered (C) Full-length HRI with the 41 identified phosphorylation sites added.
