# Supplementary material for: Structural insights into allosteric inhibition of HRI kinase by heme binding via HDX-MS
Source: Biochem J. 2025 Jun 17;482(12):859–75. doi: 10.1042/BCJ20253072 (PMC12235045; doi:10.1042/BCJ20253072)
Supplement: Online supplementary figure 3 [file bcj-482-12-BCJ20253072-supp3.pdf]

```
sp|P19525|E2AK2_HUMAN      GLRNNQRKAKRSLAPRFDLPDMKETKYTVDKRFGMDFKEIELIGSGGFGQVFKAKHRIDG   290 PKR
sp|Q9NZJ5|E2AK3_HUMAN      --TENKYDSVSGEANDSSWNDIKNSG--YISRYLTDFEPIQCLGRGGFGVVFEAKNKVDD   616 PERK
sp|Q9P2K8|E2AK4_HUMAN      RL---------------PSAAFFSETQRQFSRYFIEFEELQLLGKGAFGAVIKVQNKLDG   613 GCN2
sp|Q9BQI3|E2AK1_HUMAN      KI---------------RSR--EVALEAQTSRYLNEFEELAILGKGGYGRVYKVRNKLDG   190 HRI
                                                         .*:  :*: :  :* *.:* * :.::::*.

sp|P19525|E2AK2_HUMAN      KTYVIKRVKYNN------EKAERFVKALAKLDHVNIVHYNGCWDGFDYDP----------   334 PKR
sp|Q9NZJ5|E2AK3_HUMAN      CNYAIKRIRLPNRELA-REKVMREVKALAKLEHPGIVRYFNAWLEAPPEKWQEKMDEIWL   675 PERK
sp|Q9P2K8|E2AK4_HUMAN      CCYAVKRIPINPASRQ-FRRIKGEVTLLSRLHHENIVRYYNAWIERHERPAGPGT-----   667 GCN2
sp|Q9BQI3|E2AK1_HUMAN      QYYAIKKILIKGATKTVCMKVLREVKVLAGLQHPNIVGYHTAWIEHVHVIQPRADR----   246 HRI
                             *.:*::           :   **. *: *.* .** *  .*

sp|P19525|E2AK2_HUMAN      -------------------------------ETSDD-----------------------   339 PKR
sp|Q9NZJ5|E2AK3_HUMAN      KDESTDWPLSSPSPMDAPS----VKIRRMDPFATKEHIEIIAPSPQRSRSFSVGISCDQT   731 PERK
sp|Q9P2K8|E2AK4_HUMAN      -------PPPDSGPLA--KDDRAARGQPASDTDGLDSVEAAAPPPILSSSVEWS-TSGER   717 GCN2
sp|Q9BQI3|E2AK1_HUMAN      ----AAIELPSLEVLSDQEEDREQCGVKNDE-SSSSSIIFAEPTPEKEKRFGESDTENQN   301 HRI
                                                              .

sp|P19525|E2AK2_HUMAN      -------------------------------------------SL---------------   341 PKR
sp|Q9NZJ5|E2AK3_HUMAN      SSSESQFSPLEFSGMDHEDISESVDAAYNLQDSCLTDCDVEDGTMDGNDEGHSFELCPSE   791 PERK
sp|Q9P2K8|E2AK4_HUMAN      SASARFPA---------------------------TGPGSSDDE-DDDEDEHGGVF----   745 GCN2
sp|Q9BQI3|E2AK1_HUMAN      NKSVKYTTNLVI-----------------------RESGELESTLELQENGLAGLS----   334 HRI

sp|P19525|E2AK2_HUMAN      -----------------ES---------------------------SDYDPENS------   351 PKR
sp|Q9NZJ5|E2AK3_HUMAN      ASPYVRSRERTSSSIVFEDSGCDNASSKEEPKTNRLHIGNHCANKLTAFKPTSSKSSSEA   851 PERK
sp|Q9P2K8|E2AK4_HUMAN      SQSFLPAS-DSESDIIFDNEDENSK---------SQNQDEDCNEKNGCHE----------   785 GCN2
sp|Q9BQI3|E2AK1_HUMAN      ASSIV------EQQLPLRRNSHLEE---------SFTSTEESSEENVNFL----------   369 HRI
                                                                           .

sp|P19525|E2AK2_HUMAN      ----------------------KNSSRSKTKCLFIQMEFCDKGTLEQWIEKRRG------   383 PKR
sp|Q9NZJ5|E2AK3_HUMAN      TLSISPPRPTTLSLDLTKNTTEKLQPSSPKVYLYIQMQLCRKENLKDWMNGRCT------   905 PERK
sp|Q9P2K8|E2AK4_HUMAN      --------------------SEPSVTTEAVHYLYIQMEYCEKSTLRDTIDQ---------   816 GCN2
sp|Q9BQI3|E2AK1_HUMAN      ----------------------GQTEAQYHLMLHIQMQLCELSLWDWIVERNKRGREYVD   407 HRI
                                                      .    *.***: *        ::

sp|P19525|E2AK2_HUMAN      ----EKLDKVLALELFEQITKGVDYIHSKKLIHRDLKPSNIFLVD-TKQVKIGDFGLVTS   438 PKR
sp|Q9NZJ5|E2AK3_HUMAN      ---IEERERSVCLHIFLQIAEAVEFLHSKGLMHRDLKPSNIFFTM-DDVVKVGDFGLVTA   961 PERK
sp|Q9P2K8|E2AK4_HUMAN      ---GLYRDTVRLWRLFREILDGLAYIHEKGMIHRDLKPVNIFLDS-DDHVKIGDFGLATD   872 GCN2
sp|Q9BQI3|E2AK1_HUMAN      ESACPYVMANVATKIFQELVEGVFYIHNMGIVHRDLKPRNIFLHGPDQQVKIGDFGLACT   467 HRI
                                        .:* :: ..: ::*.  ::****** ***:    . **:*****.

sp|P19525|E2AK2_HUMAN      LKND------GK-------------RTRSKGTLRYMSPEQIS--SQDYGKEVDLYALGLI   477 PKR
sp|Q9NZJ5|E2AK3_HUMAN      MDQD------EEEQTVLTPMPAYARHTGQVGTKLYMSPEQIH--GNSYSHKVDIFSLGLI   1013 PERK
sp|Q9P2K8|E2AK4_HUMAN      HLAFSADSKQDDQTGDLIKSDPSGHLTGMVGTALYVSPEVQGSTKSAYNQKVDLFSLGII   932 GCN2
sp|Q9BQI3|E2AK1_HUMAN      DILQKNTDWT-NRNG-----KRTPTHTSRVGTCLYASPEQLE--GSEYDAKSDMYSLGVV   519 HRI
                                      .              *   **  * ***      . *. : *:::**::

sp|P19525|E2AK2_HUMAN      LAEL-LHVCDTAFETSKFFTDLRDGII---SDIFDKK----EKTLLQKLLSKKPEDRPNT   529 PKR
sp|Q9NZJ5|E2AK3_HUMAN      LFEL-LYPFSTQMERVRTLTDVRNLKF---PPLFTQKY-PCEYVMVQDMLSPSPMERPEA   1068 PERK
sp|Q9P2K8|E2AK4_HUMAN      FFEMSYHPMVTASERIFVLNQLRDPTSPKFPEDFDDGEHAKQKSVISWLLNHDPAKRPTA   992 GCN2
sp|Q9BQI3|E2AK1_HUMAN      LLEL-FQPFGTEMERAEVLTGLRTGQL---PESLRKRC-PVQAKYIQHLTRRNSSQRPSA   574 HRI
                           : *:      *  *    :. :*          : .     :   :. :   .  .** :

sp|P19525|E2AK2_HUMAN      SEILRTLTVWKK-SPEKN----------------ER----------HTC-----------   551 PKR
sp|Q9NZJ5|E2AK3_HUMAN      INIIENAVFEDLDFPGKTVLRQRSRSLSSSGTKHSRQS-----NNSHSPL----------   1113 PERK
sp|Q9P2K8|E2AK4_HUMAN      TELLKSELLPPPQMEESELHEVLHHTLTNVDGKAYRTMMAQIFSQRISPAIDYTYDSDIL   1052 GCN2
sp|Q9BQI3|E2AK1_HUMAN      IQLLQSELFQNSGNVNLTLQMK-------------------IIEQEKE-IAELKKQLNLL   614 HRI
                            :::..  .
```

. = Weak Conservation (< 0.5 Gonnet PAM 250 matrix)
: = Strong Conservation (> 0.5 Gonnet PAM 250 matrix)
* = Fully Conserved

Supplementary Figure 3

Supplementary Figure 3: Alignment of the kinase domains of all the human eIF2alpha kinases (PKR, PERK, HRI and GCN2).
