# Supplementary material for: Structural insights into allosteric inhibition of HRI kinase by heme binding via HDX-MS
Source: Biochem J. 2025 Jun 17;482(12):859–75. doi: 10.1042/BCJ20253072 (PMC12235045; doi:10.1042/BCJ20253072)
Supplement: Online supplementary figure 4 [file bcj-482-12-BCJ20253072-supp4.pdf]

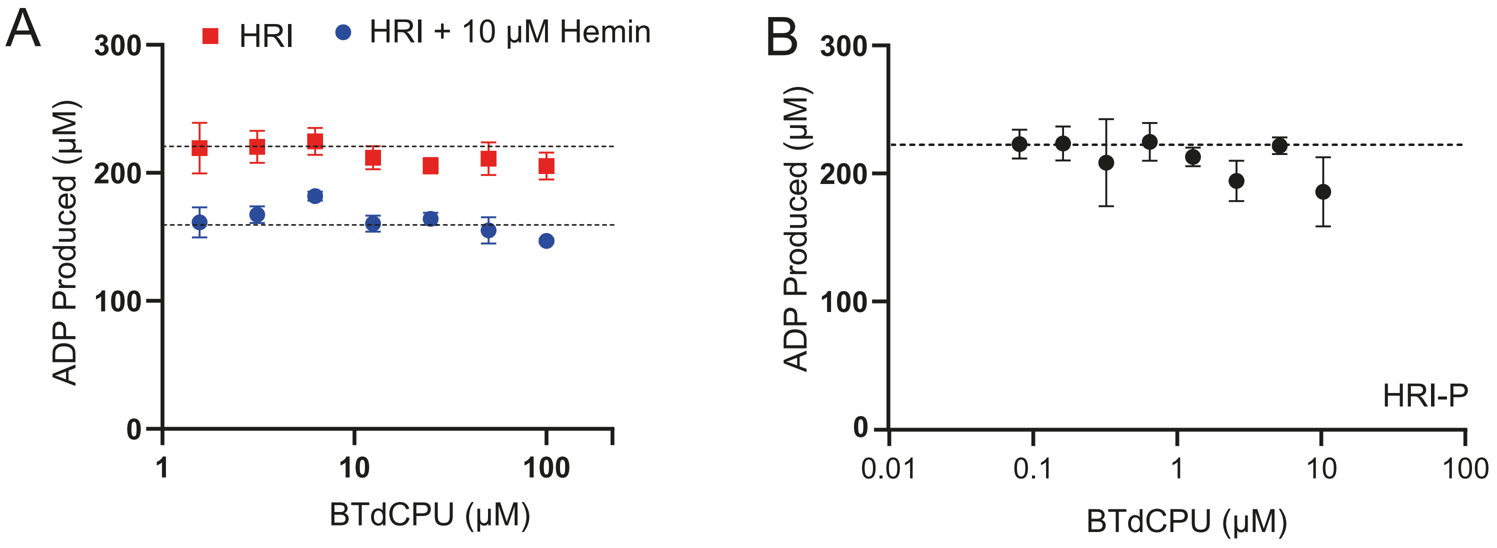

Supplementary Figure 4: BTdCPU kinase assays. (A) Activity of HRI (as measured by ADP production) on the addition of increasing BTdCPU in either the absence or presence of 10 μM Hemin. 100 nM HRI was incubated with 5 μM eIF2α and 100 μM ATP – no activation or inhibition was observed. (B) Autophosphorylated HRI (i.e. HRI pre-incubated with ATP and subsequently purified) was exposed to increasing BTdCPU in the presence of eIF2α. ADP levels were measured using ADP-GLO and plotted as a % of APO HRI-P levels.
