# Supplementary material for: Structural insights into allosteric inhibition of HRI kinase by heme binding via HDX-MS
Source: Biochem J. 2025 Jun 17;482(12):859–75. doi: 10.1042/BCJ20253072 (PMC12235045; doi:10.1042/BCJ20253072)
Supplement: Online supplementary figure 5 [file bcj-482-12-BCJ20253072-supp5.pdf]

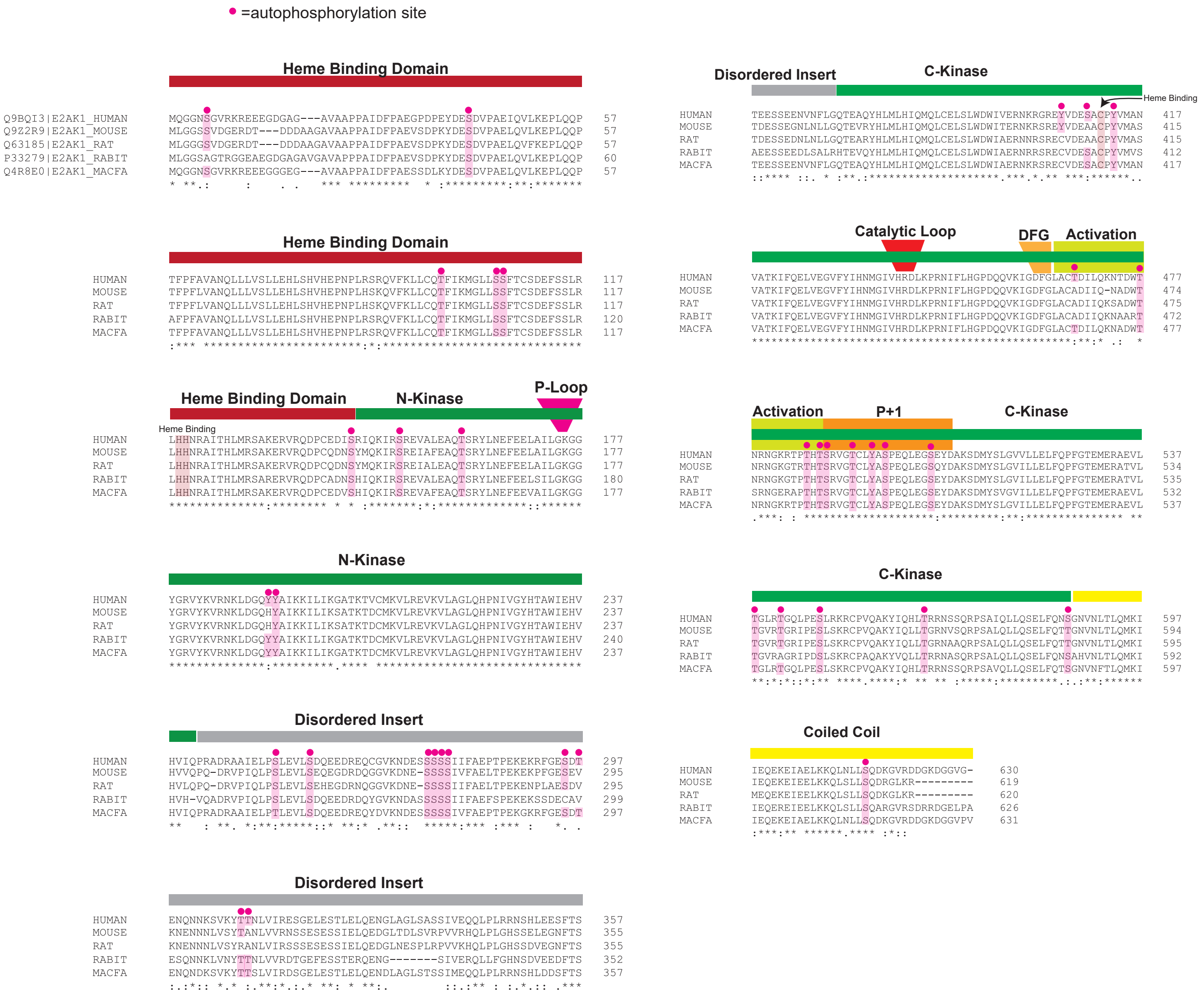

Supplmentary figure 5

Supplementary Figure 5: Species alignment of HRI. Human (H. sapiens), Mouse (M. musculus), Rat (R. ratus), Rabbit (Oryctolagus cuniculus) and Crab-eating macaque (Macaca fascicularis) of EIF2AK1 (HRI). *= conserved residues, := partial conservation. Sites of autophosphorylation in human HRI identified in this study are identified by pink highlighting.
