# Supplementary figures and images for: Structural insights into allosteric inhibition of HRI kinase by heme binding via HDX-MS
Source: Biochem J. 2025 Jun 17;482(12):859–75. doi: 10.1042/BCJ20253072 (PMC12235045; doi:10.1042/BCJ20253072)

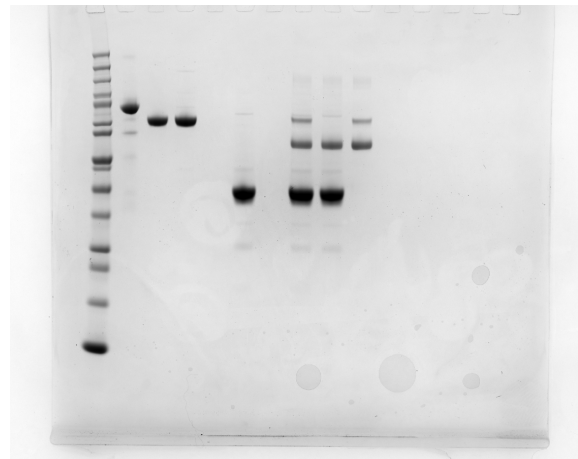

Figure 1A

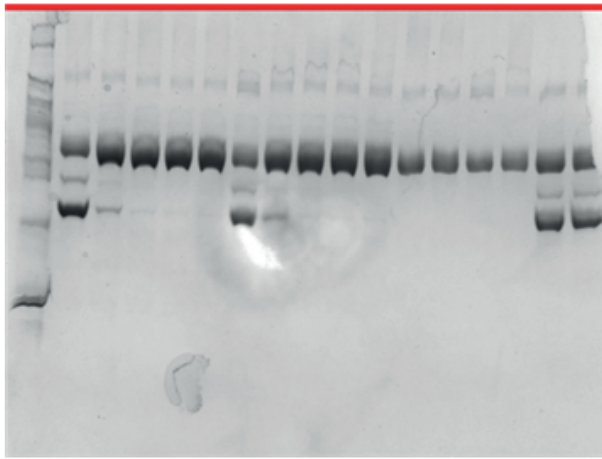

Figure 1E

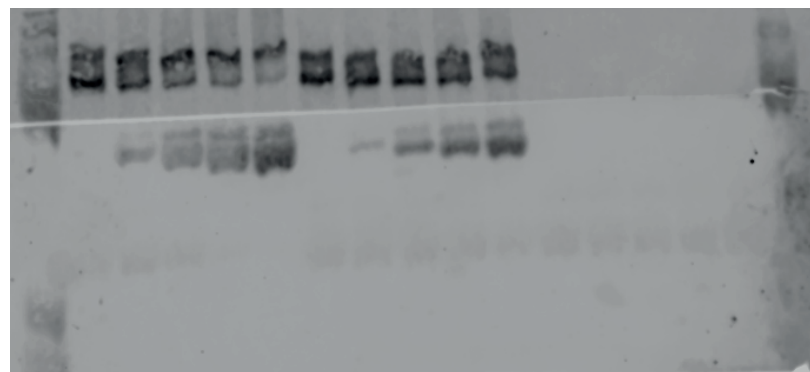

Figure 2B

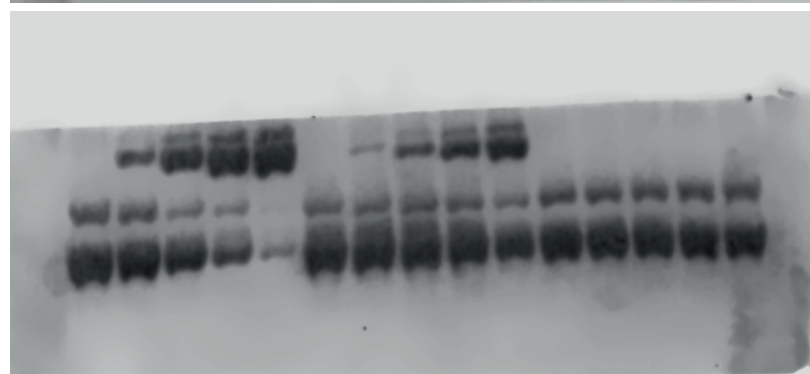

Figure 2B

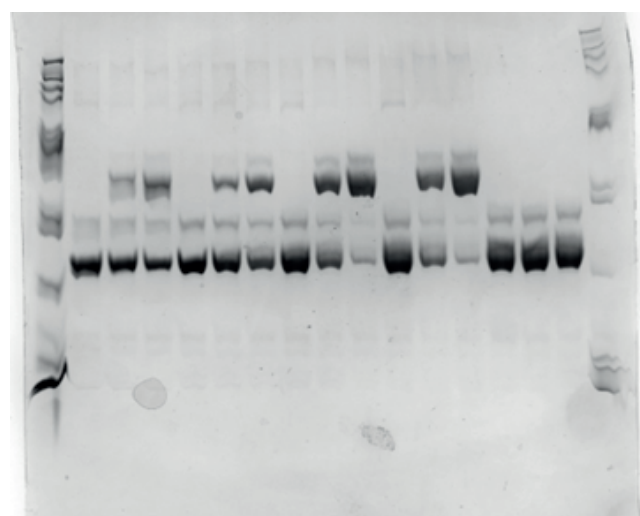

Figure 2C

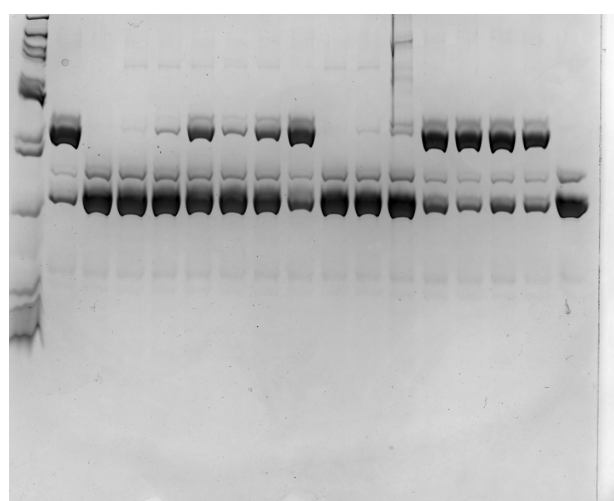

Figure 2D

Supplement: Online supplementary material [file bcj-482-12-BCJ20253072-supp6.pdf]
